# On body and off body communication using a compact wideband and high gain wearable textile antenna

**DOI:** 10.1038/s41598-024-64932-6

**Published:** 2024-06-24

**Authors:** Deepti Sharma, Sachin Kumar, Rakesh Nath Tiwari, Hyun Chul Choi, Kang Wook Kim

**Affiliations:** 1 Department of Electronics and Communication Engineering, GL Bajaj Institute of Technology and Management, Greater Noida, Uttar Pradesh 201306 India; 2 Department of Electronics and Communication Engineering, Galgotias College of Engineering and Technology, Greater Noida, Uttar Pradesh 201310 India; 3 Department of Electronics and Communication Engineering, Madanapalle Institute of Technology and Science, Madanapalle, Andhra Pradesh 517325 India; 4 School of Electronic and Electrical Engineering, Kyungpook National University, Daegu, 41566 Republic of Korea

**Keywords:** Engineering, Materials science

## Abstract

In this paper, a compact low-profile dual-band wearable textile antenna is proposed for on-body and off-body communications. The presented antenna works efficiently in the 5G n79 frequency band (4.4 − 5 GHz) and the ISM band (5.725 − 5.875 GHz). The designed antenna has an ultra-wide impedance bandwidth of 2.01 GHz and peak realized gains of 10.5 dBi and 12 dBi at 4.5 GHz and 5.8 GHz, respectively. The antenna has a small footprint (π × 0.3*λ*_0_^2^), which is inspired by circular fractal geometry. The performance of the presented wearable antenna is evaluated at various body parts, including the arm, wrist, and chest. The link margin is evaluated in the on-body and off-body communication scenarios, i.e., communication with the implantable antenna and the outside-body antenna, which is 80 dB and 65 dB at 4.5 GHz and 5.8 GHz, respectively. The 1 gm/10 gm specific absorption rate values at 4.5 GHz and 5.8 GHz are 0.12/0.098 and 0.11/0.082, respectively, which are significantly lower than the standard values, making the proposed antenna suitable for modern wearable applications.

## Introduction

**Figure 1:**
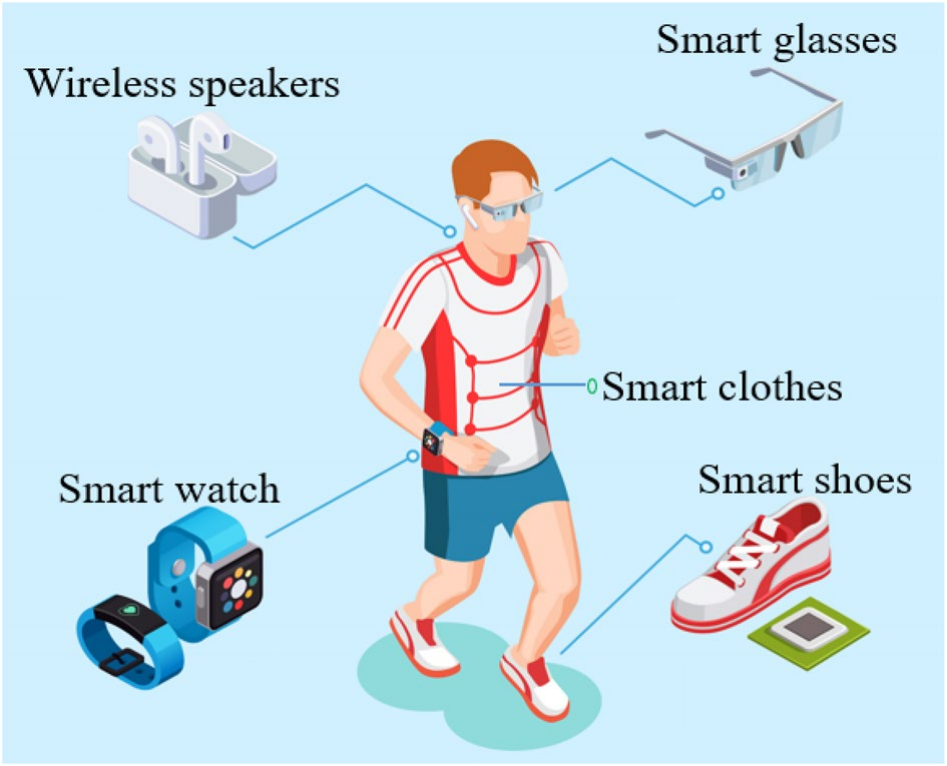
Schematic of a human with multiple smart wearable devices (human body figure is adapted from^7^).

Body area network (BAN) is currently being used in various industries including healthcare, military, sports, and firefighting. BAN employs a wide range of smart devices, such as smart glasses, smart watches, healthcare bands, smart shoes, glucose monitoring systems, etc., for a variety of applications, as illustrated in Fig. 1. Also, each device needs to have a wearable antenna that can communicate with implantable medical devices and external body devices. Wearable antennas are challenging to design as they must withstand various environmental, temperature, and bending changes. In addition, the material of the wearable antenna must be flexible and breathable to maintain wearing comfort, and textile antennas are the best choice in this regard. Since wearable antennas operate on the human body, their frequency of operation and bandwidth are influenced by the loading effect of the human body. To avoid such conditions, the wearable antenna should not come into direct contact with the human body, as direct contact increases the harmful radiation effects on the body.

In the literature, various types of textile and wearable antennas have been presented. A rectangular ring-shaped antenna with a size of 50 mm × 50 mm was reported^1^. The impedance bandwidth of the reported antenna was narrow for both 2.4/5.2 GHz WLAN bands, and the gain was −3.2/6.6 dBi. A proximity-fed textile-based single band (2.45 GHz) antenna was reported with a size of 0.43*λ*_0_ × 0.43*λ*_0_ and peak gain and impedance bandwidth of 6.3 dBi and 6.6%, respectively^2^. A textile-based multi-layer antenna with an area of 0.49*λ*_0_ × 0.49*λ*_0_ was discussed in^3^, which operated in the 2.45 GHz and 3.45 GHz frequency bands with 6.7 dBi and 8.9 dBi gain values, respectively. However, the antennas reported in^2^ and^3^ were not low-profile as they had multi-layer structures, and the antenna presented in^2^ only worked in one frequency band, whereas wearable antennas need to perform on-body and off-body communications, so dual-band operation is required. In^4^, a multiband textile antenna with narrow impedance bandwidths and gain values of −0.81/ −2.81/ −1.16/2.8 dBi in the operating frequency bands of 2.4/3.32/3.9/5.8 GHz was presented. However, wide impedance bandwidths are required for wearable antennas, making the reported antenna immune to detuning caused by bending or other reasons due to wearer movement.

A monopole textile antenna was proposed in^8^, which had a high gain value of 7.2 dBi, and it works only in the single ISM 2.45 GHz frequency band. A wearable substrate integrated waveguide (SIW) antenna for wrist wearable applications in the 2.45 GHz was discussed^9^, with a peak antenna gain of 3.74 dBi. A single band (8 GHz) antenna was proposed for military applications^5^, with a peak gain of 5.2 dBi but it had larger footprints. A multiband textile antenna with an ultra-wideband and a gain of 7.2 dBi was proposed^6^. However, specific absorption rate (SAR) analysis was not performed in the presented work, which is an important parameter in the design of wearable antennas as it defines the maximum value of input power that can be safely handled by the human body. A dual ISM-band planar inverted F-antenna (PIFA) working at 433 MHz and 2.4 GHz was discussed in^10^. The impedance bandwidths at 433 MHz and 2.45 GHz were 8.0% and 12.6%, respectively, with on-body gains of −0.6 dBi and 6.8 dBi. A high-profile PIFA antenna with an impedance bandwidth of 10% and a peak gain of 6.7 dBi was reported in^11^, with a size of 0.77*λ*_0_ × 0.51*λ*_0_ and made entirely of textile material. A low-profile dual-band wearable antenna with peak gains of 2.9 dBi in the 2.4 GHz band and 4.2 dBi in the 4.5 GHz frequency band was proposed^12^. However, it will be difficult to integrate into clothing as this antenna was made of polyamide material. A comparison of various textile antennas proposed for wearable applications is provided in Table 1. When all of the above-mentioned textile antennas are compared, it can be concluded that there is a need for a multiband antenna that can perform on-body and off-body communications while having a wide impedance bandwidth, high gain, and small footprints. Keeping all of the mentioned considerations, this paper proposes a low-profile compact and dual-band (5G n79 and ISM 5.8 GHz) antenna for on-body and off-body communications. The proposed antenna is designed on a multi-layer cylindrical phantom using a modified circular fractal approach, which resulted in a small footprint of 0.28*λ*_0_^2^. The circular loop helps to confine the fields to the antenna geometry, allowing for high gain values of 10.5 dBi and 12 dBi in the 4.5 GHz and 5.8 GHz bands, respectively, as well as low SAR values. To demonstrate the practicality of the antenna, the link margin of the proposed wearable antenna is calculated by communicating with the implantable antenna and the outside-body antenna.

**Table 1:** Comparison of the proposed antenna with other reported textile antennas.

[Ref.] (Year)	^2^ (2020)	^3^ (2021)	^4^ (2022)	^5^ (2022)	^6^ (2023)	Prop.
Antenna type	Textile	Textile	Textile	Textile	Textile	Textile
Dielectric constant (*ε*_*r*_)/loss tangent (*δ*)	1.7/0.008	2.2/0.0009	1.72/0.045	1.4/0.02	1.06/0.0001	1.72/0.04
Dielectric constant (*ε*_*r*_)/loss-tangent (*δ*)/thickness measurement technique	–	–	Resonance method/microstrip ring resonator method	–	–	RF impedance/material analyzer/digital vernier gauge
Profile	Multi-layer	Multi-layer	Single layer	Single layer	Single layer	Single layer
Area (mm^2^)	53 × 53	60 × 60	60 × 60	55 × 40	84 × 69	π × (12.5)^2^
Area (*λ*_0_^2^)	0.43 × 0.43 (0.18)	0.49 × 0.49 (0.24)	0.64 × 0.64 (0.40)	1.46 × 1.06 (1.54)	0.55 × 0.67 (0.36)	π × (0.3)^2^ (0.28)
Frequency (GHz)	2.4	2.45/3.45	2.4/3.32/3.93/5.8	8	2.4/5	4.5/5.8
Bandwidth (%)	6.6	4.9/6.7	3.7/5.7/ 5.8/9.8	13.1	5/76	44/35
Peak gain (dBi)	6.3	6.7/8.9	−0.81/ −2.81/ −1.16/2.8	5.2	7.2	10.5/12.0
Bending analysis done	Yes	Yes	No	Yes	Yes	Yes
SAR (W/Kg) 1 g/10 g	2.7285/3.3641 (at 1 W)	0.1/0.04 (at 0.5 W)	0.11/0.33 (at 1 W)	0.7/– (at 1 W)	–	0.12/0.098 0.11/0.082 (at 1 W)

## Methodology

### Antenna design

**Figure 2:**
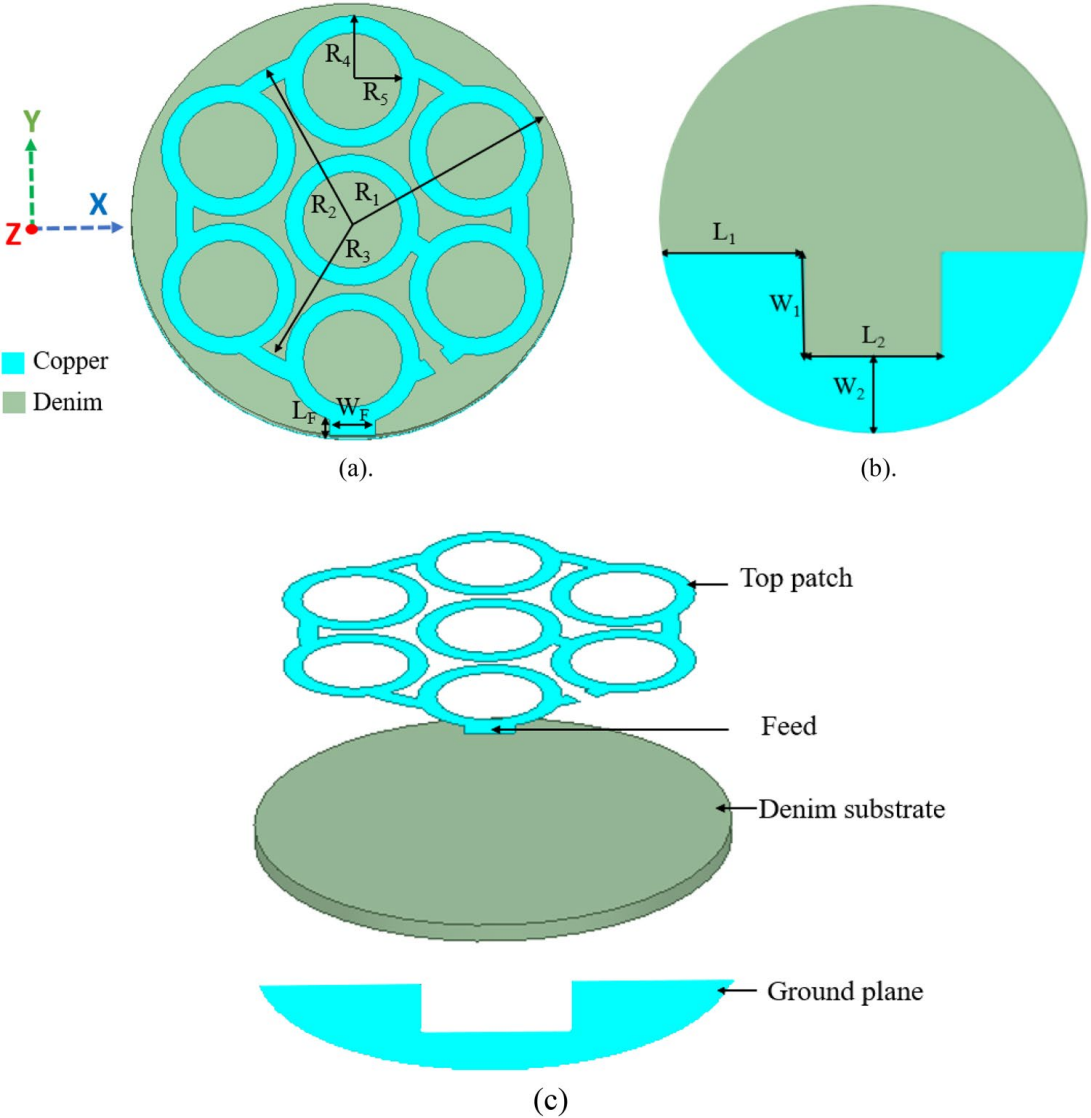
Design of the proposed wearable antenna: (**a**) Top, (**b**) Bottom, (**c**) Exploded view (*R*_1_ = 12.5 mm, *R*_2_ = 10 mm, *R*_3_ = 9 mm, *R*_4_ = 3.8 mm, *R*_5_ = 2.8 mm, *L*_*F*_ = 1 mm, *W*_*F*_ = 2.6 mm, *L*_1_ = 8.25 mm, *L*_2_ = 8 mm, *W*_1_ = 6 mm, *W*_2_ = 4.5 mm).

The proposed dual-band antenna is designed on the denim substrate of dielectric constant (*ε*_*r*_) of 1.72, loss tangent (*δ*) of 0.04, and a thickness of 1 mm. The dielectric constant and loss tangent of the substrate material are characterized using a radio frequency impedance/material analyzer (Agilent E4991A), and the thickness of the denim substrate is measured using a digital vernier gauge. Copper is used as a conducting material for the proposed textile antenna as it provides good impedance matching, gain, and efficiency^13^. The EM tool Ansys HFSS® is used to design and implement the proposed textile antenna. The antenna’s design and dimensions are shown in Figs. 2(a–c), and its overall size is π × (12.5 mm)^2^. A modified circular fractal geometry is used to design the antenna and a partial slotted ground plane is designed on the back side. The antenna works in dual frequency bands: 5G n79 and ISM 5.8 GHz with ultra-wide bandwidth. A fractal outline is chosen to design the proposed antenna because fractal geometry is an extension of conventional geometry^14,15^. Its introduction offers designers and engineers the extraordinary prospect of exploring a virtually unlimited number of previously unavailable configurations for possible use in the development of new and innovative antenna designs^16,17^. In recent years, many research groups have successfully used fractal geometries to realize miniaturised, multiband, and wideband antennas^18–20^. In the proposed work, a modified circular fractal geometry is used to achieve dual-frequency bands with ultra-wide bandwidth.

**Figure 3:**
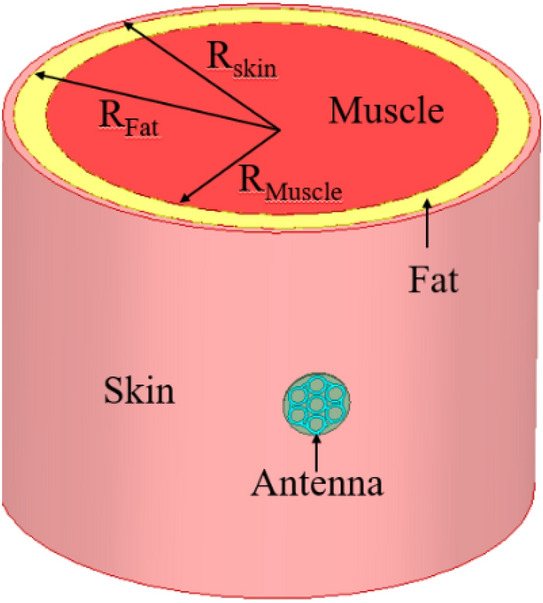
Antenna design on the multi-layer cylindrical chest phantom body model (*R*_Skin_ = 100 mm, *R*_Fat_ = 96 mm, *R*_Muscle_ = 84 mm).

The proposed antenna is inspired by fractal geometry, in which antenna design begins with the mapping of a single large circle into seven smaller circles of one-third diameter. This change lowers the resonant frequency while also enabling dual-band behaviour at 4.4 GHz and 9.3 GHz. Next, the diameter of each circle is reduced from 4 to 3.8 mm and connected with the split ring, which helps to create the 4.5 GHz and 5.8 GHz bands. Finally, all of the small circles are converted into circular rings of 1 mm width, resulting in a wide bandwidth of 2.01 GHz in the reported resonating frequency bands. Furthermore, the antenna is designed on a multi-layer (skin-fat-muscle) cylindrical chest phantom (150 mm length) for wearable on-body and off-body communication, as shown in Fig. 3. The dielectric properties of each layer of the chest phantom are taken from^21^.

### Evolution of the implantable antenna

**Figure 4:**
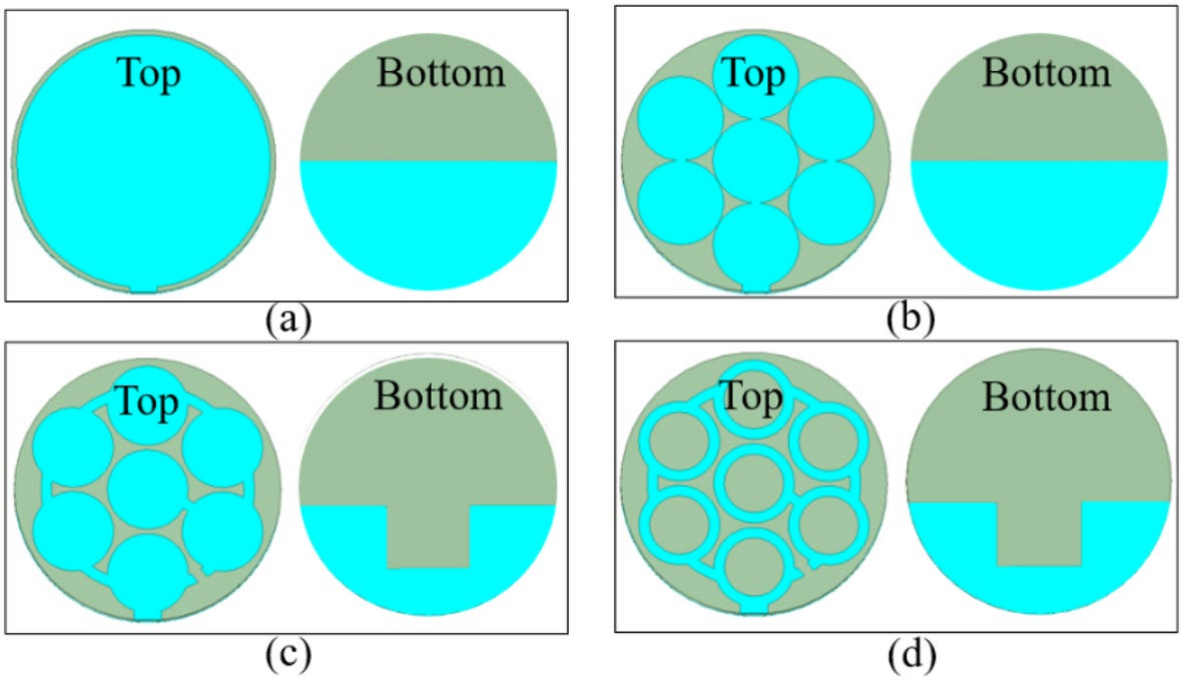
Evolution of the antenna design: (**a**) First step (step 0), (**b**) Second step (step 1), (**c**) Third step (step 2), (**d**) Fourth step (step 3).

**Figure 5:**
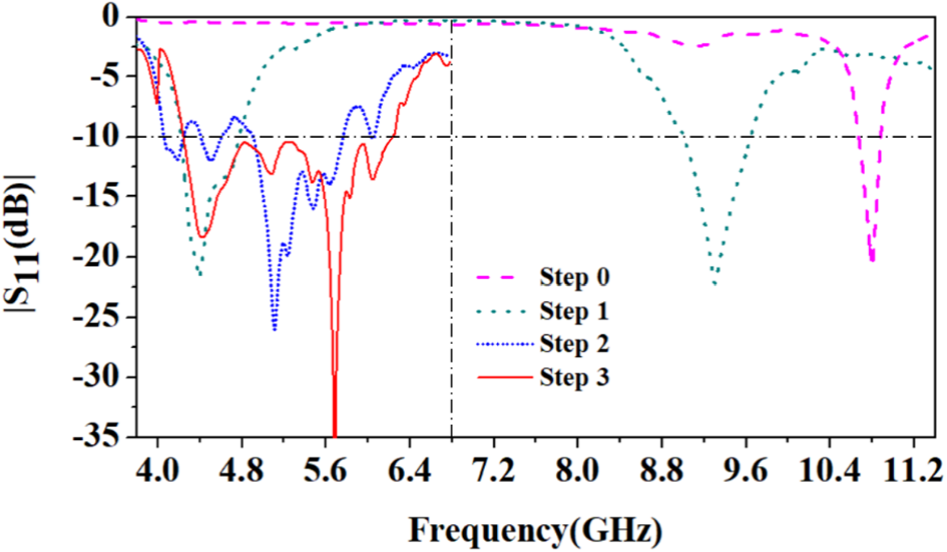
S_11_-parameters correspond to the antenna evolution.

**Figure 6:**
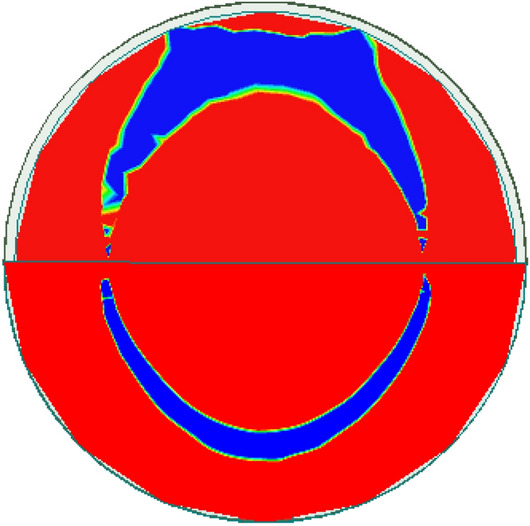
Electric field intensity at 10.8 GHz in step 0 of the antenna evolution.

Figure 4 depicts the evolution of antenna design. Initially, in step 0, a simple circular patch antenna with a radius of 12.5 mm is designed on the denim substrate, with a partial ground plane of 12.5 mm in width on the backside. A partial ground plane instead of a full ground plane leads to a wider impedance bandwidth and high-efficiency^22,23^. The antenna resonates at 10.8 GHz frequency and has an impedance bandwidth of 300 MHz, as shown in Fig. 5. At 10.8 GHz, the antenna has two nulls, as shown in Fig. 6, which corresponds to the TM_11_ mode.

**Figure 7:**
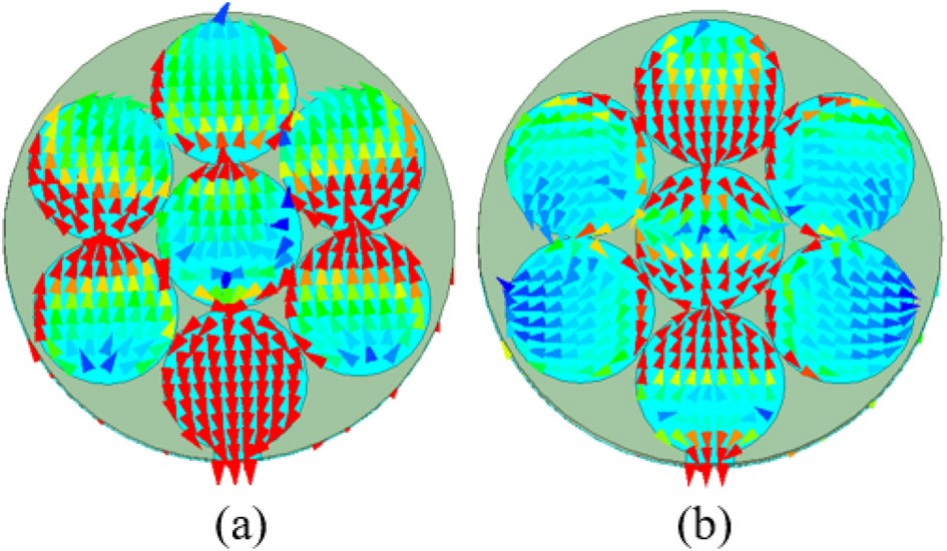
Current distribution in step 1: (**a**) 4.4 GHz, (**b**) 9.3 GHz.

In the next step (step 1), the fractal approach is considered, in which the diameter of one large circle (12 mm) is replaced by three small circles of a diameter of 4 mm. The total number of seven circles can be fit into the one big circle of step 1. And each small circle of step 1 has one-third of the diameter (4 mm) of a big circle of step 0. In this mapping of the circle, the current travels a little longer and has two different paths than step 0, therefore, the antenna is resonating in dual frequency bands: at 4.4 GHz and 9.3 GHz, as shown in Fig. 5. As per the current distribution of step 1 of Fig. 7, it can be observed that at 4.4 GHz, the whole patch is activated with high current density, but, at 9.3 GHz, three small circles at the center of the geometry have high current density. In this step, it can be noticed that the frequency band at 4.4 GHz resonant frequency is useful in the 5G n79 frequency band (4.4 − 5 GHz). However, the 9.3 GHz frequency band needs to be tuned to a useful band.

Therefore, in the third step (step 2), the radius of each circle is reduced by 0.2 mm and connected using a circular ring of diameter 10 mm and width of 1 mm. The ring connecting all the small circles except the middle one is split at the right side of the first circle (connected to the microstrip feed). The circle with the disconnected ring is connected to the center circle by a small 1 mm wide metal strip. The ground plane width is also reduced, which is now 10.5 mm and a rectangular slot of 8 mm × 6 mm is also etched to broaden the bandwidth. This change is done to increase the current path to shift the 9.3 GHz resonance to the lower side. Now, two close resonances with increased bandwidths at 4.5 GHz and 5.1 GHz are observed but matching in the 5G 4.5 GHz frequency band deteriorated.

In the last step (step 3), all the small circles are replaced by circular rings of 1 mm thickness. Due to this, current is confined to the periphery of the circular rings, and a better impedance matching is achieved at the 4.5 GHz and 5.8 GHz frequency bands with a wide bandwidth of 2.01 (4.24 − 6.25) GHz.

**Figure 8:**
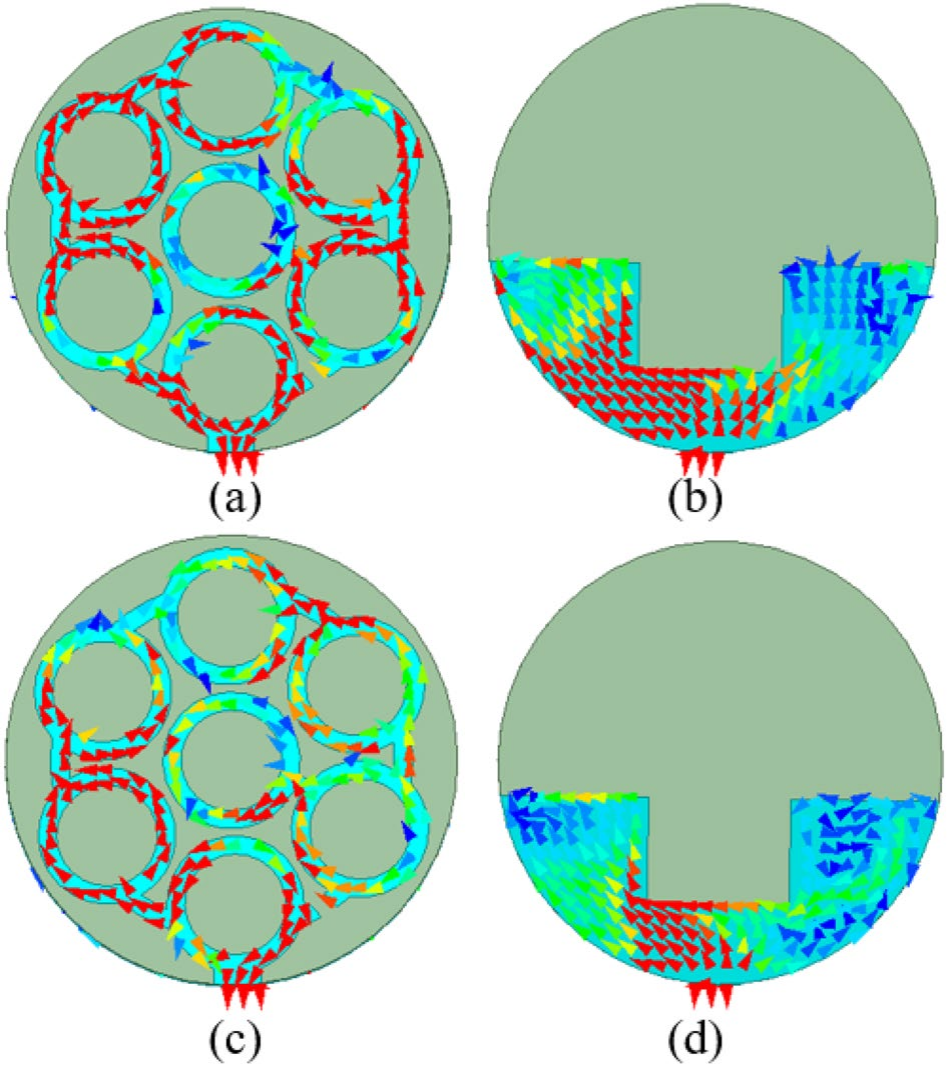
Current distribution in step 3 (proposed antenna): (**a**) Top patch at 4.5 GHz, (**b**) Ground plane at 4.5 GHz, (**c**) Top patch at 5.8 GHz, (**d**) Ground plane at 5.8 GHz.

The current distributions of the proposed antenna, at 4.5 GHz and 5.8 GHz, are shown in Fig. 8. It can be seen that both the ground plane and the circular ring geometry play an equally important role in both frequency bands, as current density is high at both frequency bands.

## Antenna analysis

### Bending analysis

**Figure 9:**
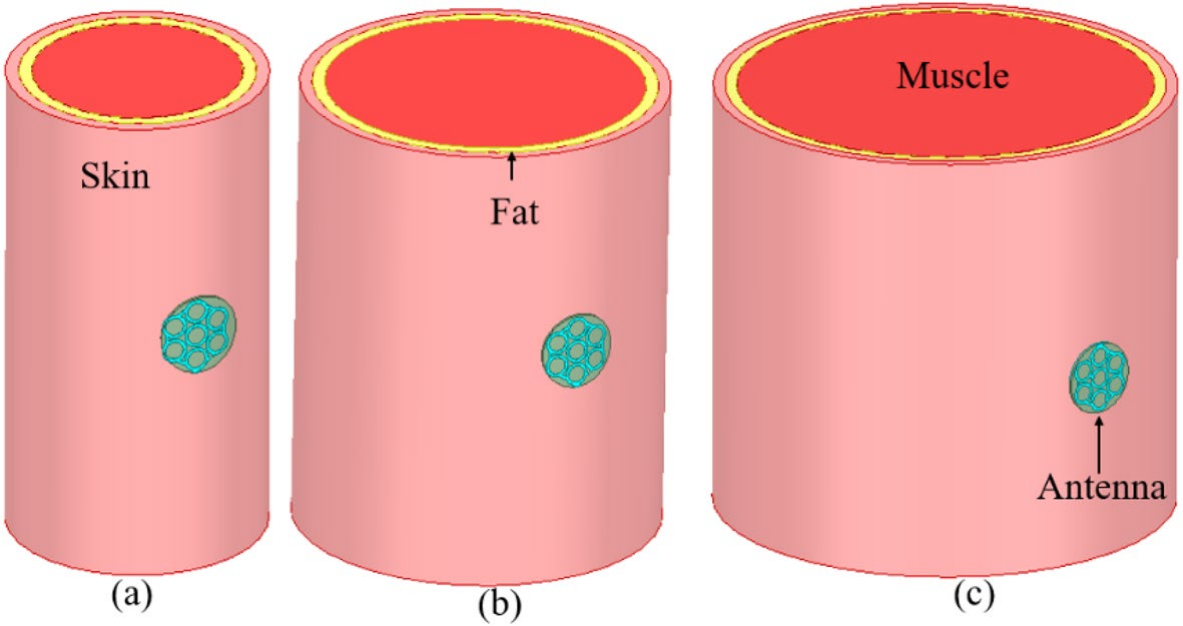
Bending of the proposed antenna on multi-layer arm phantom body model of radius: (**a**) 40 mm, (**b**) 60 mm, (**c**) 80 mm.

**Figure 10:**
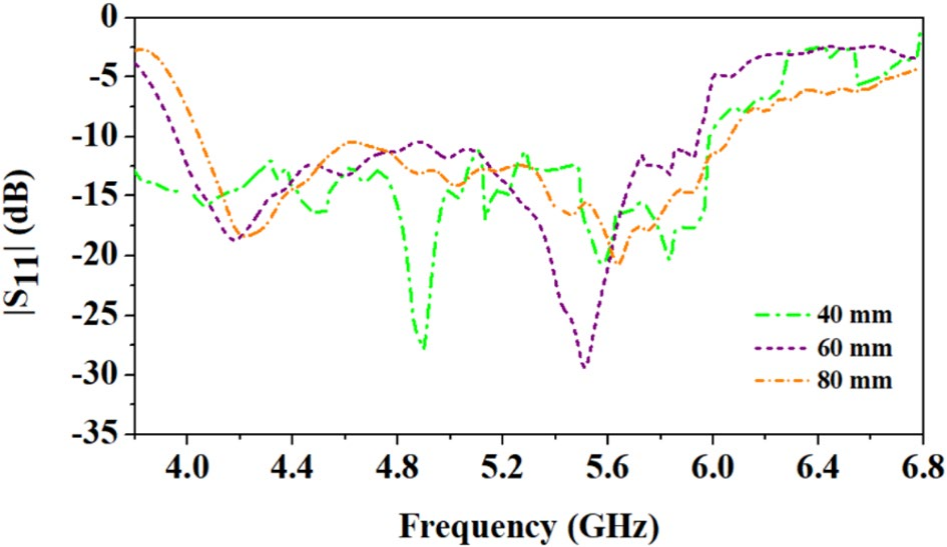
Comparison of reflection coefficients of the proposed antenna when placed on a multi-layer phantom body model of different radii (40 mm, 60 mm, and 80 mm).

Since wearable antennas operate on the human body and can bend. Therefore, bending analysis of the proposed antenna at different radii (40 mm, 60 mm, and 80 mm) is performed, as shown in Fig. 9, and the corresponding reflection coefficients are shown in Fig. 10. It can be observed from Figs. 9 and 10 that the antenna suffers more bending at the smallest radius (40 mm) and the resonant frequency shifts towards the lower side. The 4.5 GHz frequency band shifts to 4.15 GHz and 5.8 GHz to 4.9 GHz with multiple adjacent resonances. As the bending radius decreases, the resonant frequency decreases^24^. However, the antenna still covers the 4.5 GHz and 5.8 GHz bands. Similarly, at the bending of 60 mm and 80 mm radius, there are little shifts, but the function of the antenna will not be affected because of the wide bandwidth. Thus, the wide impedance bandwidth of the antenna is important when it faces conditions that can cause detuning.

### Gain analysis at different gaps between antenna and multi-layer human body model

**Figure 11:**
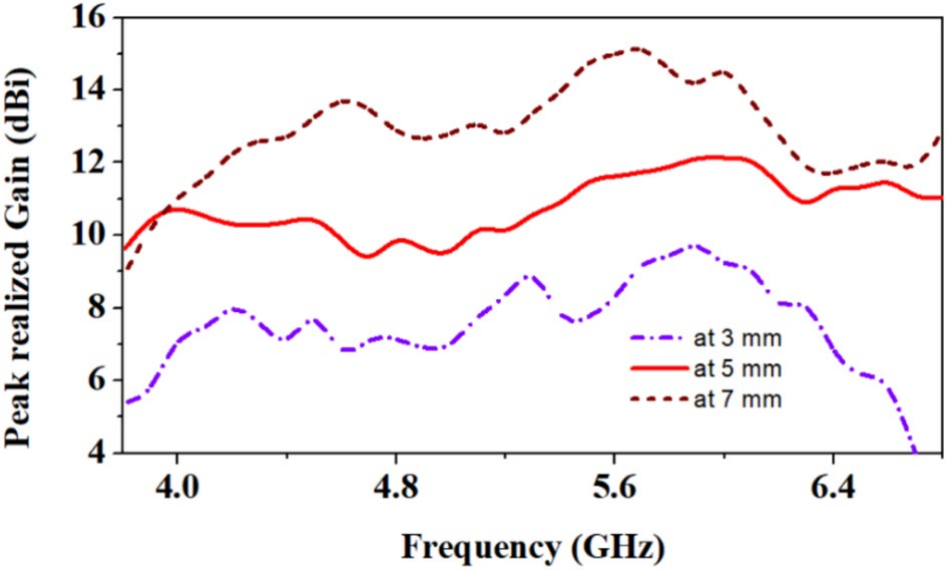
Comparison of the peak realized gain at different values of the gap of the antenna to the human body.

In this section, the gain of the antenna is analyzed, when placed at different distances from the multi-layer phantom human body model in the simulation. For the analysis purpose, distances of 3 mm, 5 mm, and 7 mm are considered. It is well known that the designed antenna should not be in direct contact with the human body because direct contact increases harmful radiation effects on the human body. Therefore, to avoid such conditions, the antenna is kept at a 5 mm distance from the human body. It is common practice in the designing of wearable antennas to keep some distance between the human body and the antenna. When the distance between the antenna and the human body model is reduced from 5 to 3 mm, the peak realized gain of the antenna is reduced from 10.5 to 7.9 dBi at 4.5 GHz and from 12 to 9.1 dBi at 5.8 GHz, as shown in Fig. 11. Similarly, when distance is increased from 5 to 7 mm, gain values increase at both the reporting frequency bands 4.5 GHz and 5.8 GHz and become 13 dBi and 14.9 dBi, respectively. Since the human body is lossy, and biological tissues have high relative permittivity and are conductive, the reduction in the gain of the antenna occurs when it is operating closer to the human body. The proposed antenna has a high gain due to its slotted ground plane and top loop-patch structure. In the literature, many researchers proposed different types of slotted ground plane to enhance the gain of the antennas^25–27^.

- **A.** **Comparison of reflection coefficients and efficiencies in on-body and off-body conditions**

**Figure 12:**
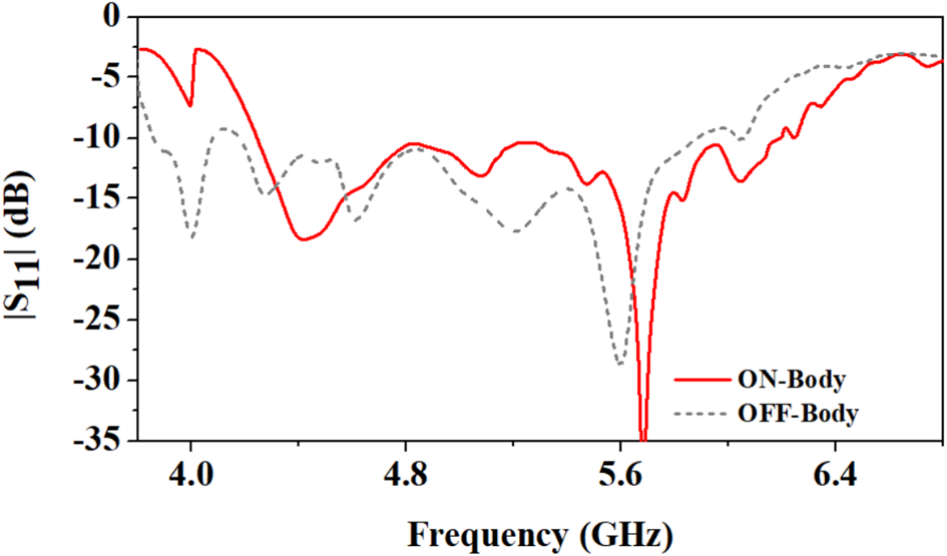
Comparison of reflection coefficients of the antenna in the on-body and off-body scenarios.

Figure 12 depicts the antenna's reflection coefficient characteristics in both on-body and off-body conditions. In off-body mode, the antenna's bandwidth is slightly wider near the lower resonance. The antenna is optimised to cover the 5G n79 frequency band (4.4–5 GHz) and ISM band (5.725–5.875 GHz) when placed on the human body.

**Figure 13:**
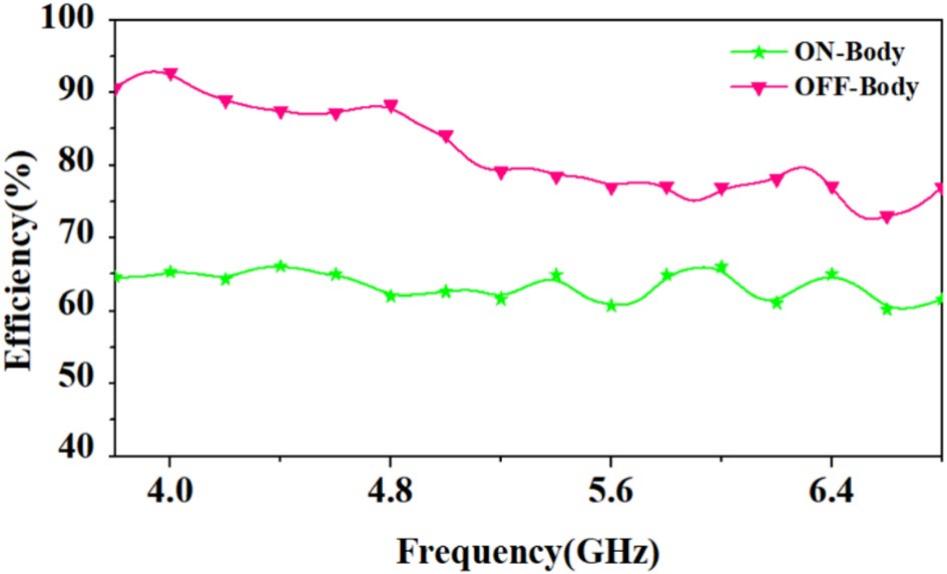
Comparison of the efficiency of the antenna in on-body and off-body scenarios.

The loop patch tends to interact less in the near-field (with the surrounding biological environment) as it is less electric and more magnetic, which can improve antenna efficiency^28^. Therefore, a loop-based patch is used in the antenna design. Figure 13 shows a comparison of the antenna's efficiency for both on-body and off-body conditions. As it is well known that the human body is lossy in nature and as the antenna is placed on the body, losses increase, so the antenna’s efficiency is reduced.

**Figure 14:**
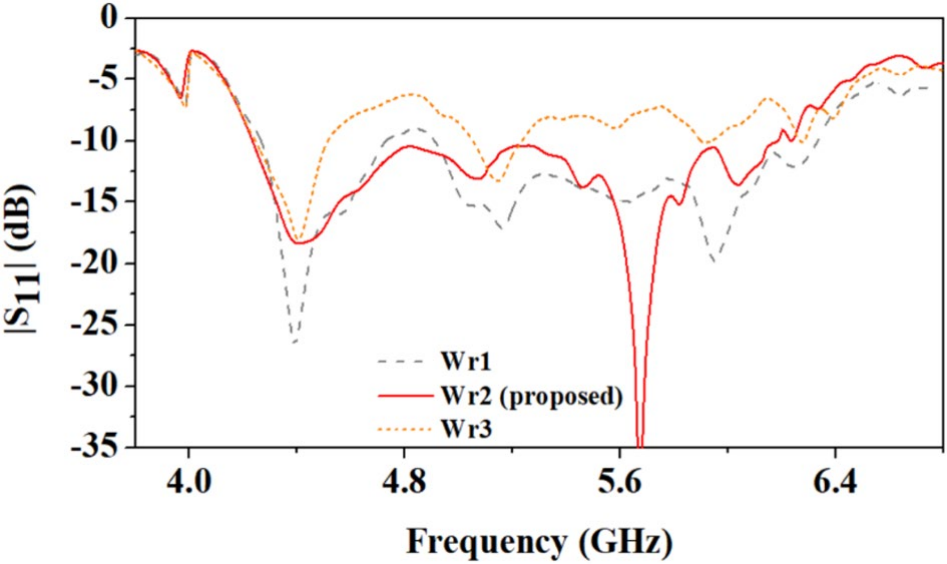
Comparison of reflection coefficients of the antenna at different widths of circular rings (*W*_*r*1_ = 0.8 mm, *W*_*r*2_ = 1.0 mm, *W*_*r*3_ = 1.2 mm).

As shown in Fig. 14, the efficiency of the antenna at 4.5 GHz in on-body and off-body scenarios is 63.6% and 87.5%, respectively. And, at 5.8 GHz, the on-body and off-body scenarios are 64.9%, and 76.4%, respectively. In the off-body case, it is important to note that the proposed antenna is more efficient at 4 GHz than at 4.5 GHz because it has better impedance matching at 4 GHz, as shown in Fig. 13.

- **B. Parametric analysis at different widths of circular rings**

In the optimization of the antenna results shown in Fig. 14, the widths of all circular rings are important. It is observed that when the widths are kept equal to 1 mm (*W*_*r*1_), a wide bandwidth of 2.01 GHz is achieved due to the merger of the 4.5 GHz and 5.8 GHz frequency bands. However, when the width is less than 1 mm (0.8 mm) or more than 1 mm (1.2 mm), the frequency bands 4.5 GHz and 5.8 GHz are separated.

- **C. Parametric analysis at different widths (*W***_**1**_**) and lengths (*L***_**2**_**) of the ground plane**

**Figure 15:**
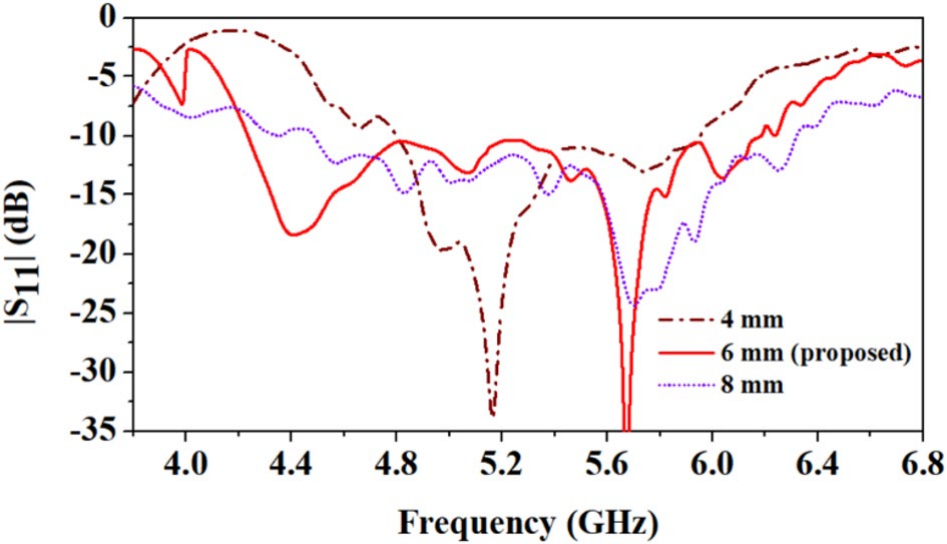
Comparison of reflection coefficients of the antenna at different widths of the ground plane slot (*W*_1_ = 4 mm, 6 mm, and 8 mm).

Figure 15 depicts the effect of the width of the rectangular ground slot (*W*_1_) on the reflection coefficients of the antenna. The antenna resonates at 4.5 GHz and 5.8 GHz with merged close resonances, resulting in a wide impedance bandwidth of 2.01 GHz. However, when the ground plane width is reduced to 4 mm, the lower resonance shifts to 5.16 GHz from 4.4 GHz, while the bandwidth of the higher resonance decreases by 1.15 GHz (4.8 − 5.95 GHz). When the width of the ground slot is increased to 8 mm, the lower frequency band 4.5 GHz detunes, and the impedance bandwidth shifts slightly to the higher side of the frequency band. It can be concluded that increasing the width of the ground slot increases bandwidth, but at the optimal ground slot width (6 mm), an appropriate tuning of the resonances 4.5 GHz and 5.8 GHz with a wide impedance bandwidth of 2.01 GHz is achieved.

**Figure 16:**
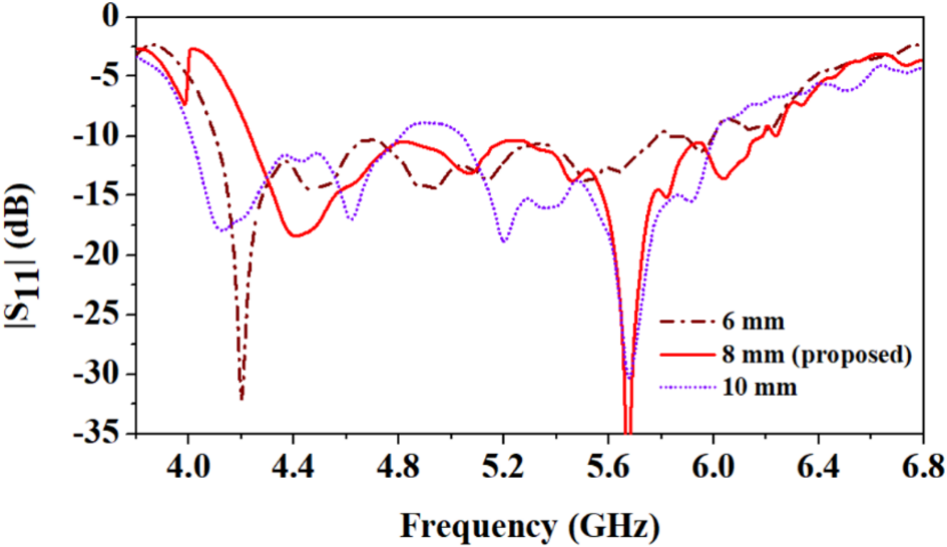
Comparison of reflection coefficients of the antenna at different lengths of the ground plane slot (*L*_2_ = 6 mm, 8 mm, and 10 mm).

Figure 16 depicts the variations in reflection coefficients caused by different lengths (*L*_2_) of the rectangular ground slot. The optimum value of the ground slot is 8 mm, at this length, both the frequency bands 4.5 GHz and 5.8 GHz are well-tuned and have a wide bandwidth. However, when the ground slot length is reduced (6 mm), the lower resonant frequency shifts to 4.2 GHz, while matching at the higher resonance deteriorates.

Furthermore, when the ground slot length is increased to 10 mm, multiple resonances are observed at 4.1 GHz, 4.6 GHz, 5.15 GHz, 5.3 GHz, 5.7 GHz, and 5.8 GHz, but the bandwidth to the higher resonance (5.8 GHz) is reduced, which is undesirable. So, the 8 mm length (*L*_2_) of the ground slot is a good choice for the proposed antenna.

## Fabrication and measurement

- **A. Measurement of S-parameters on different parts of the human body**

**Figure 17:**
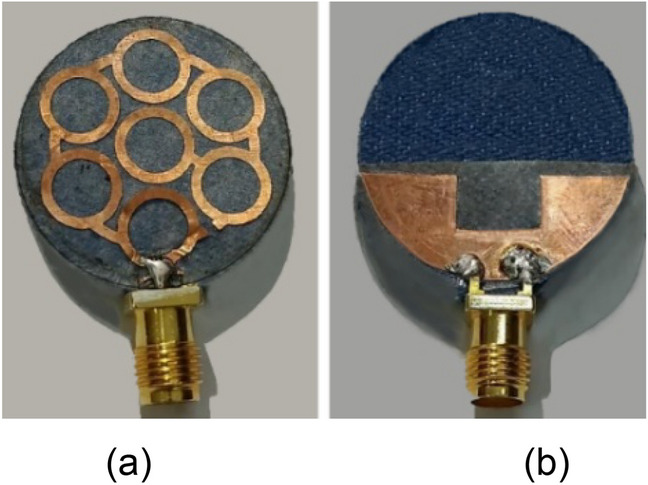
Fabricated prototype of the antenna: (**a**) Top, (**b**) Bottom.

**Figure 18:**
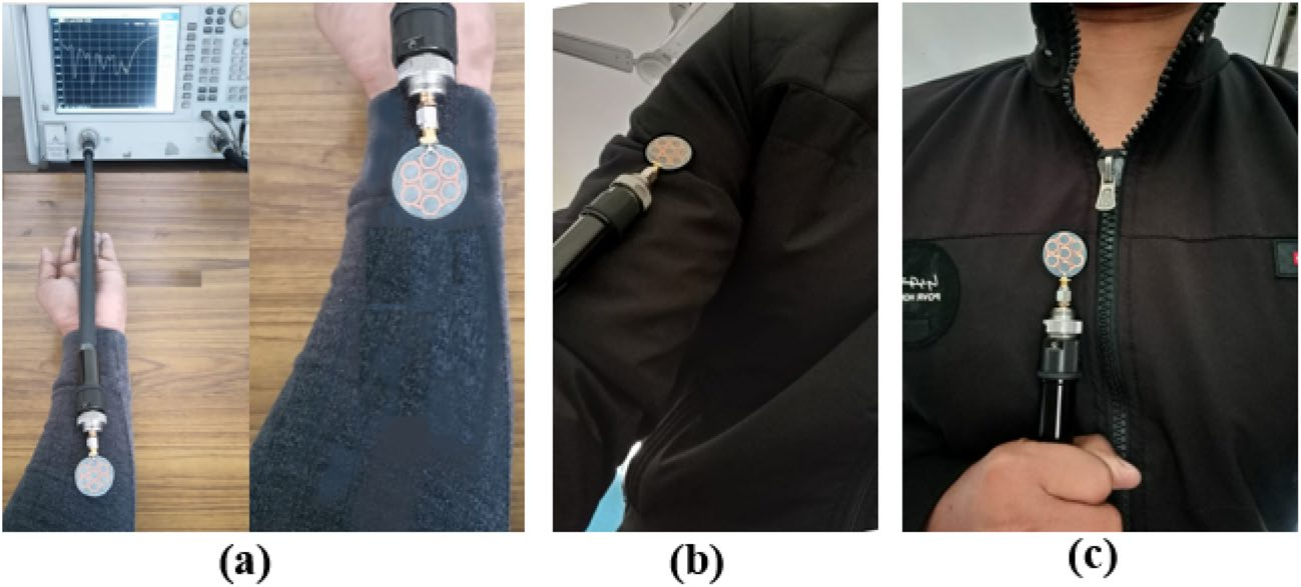
Proposed antenna measurement on (**a**) Wrist, (**b**) Arm, and (**c**) Chest.

**Figure 19:**
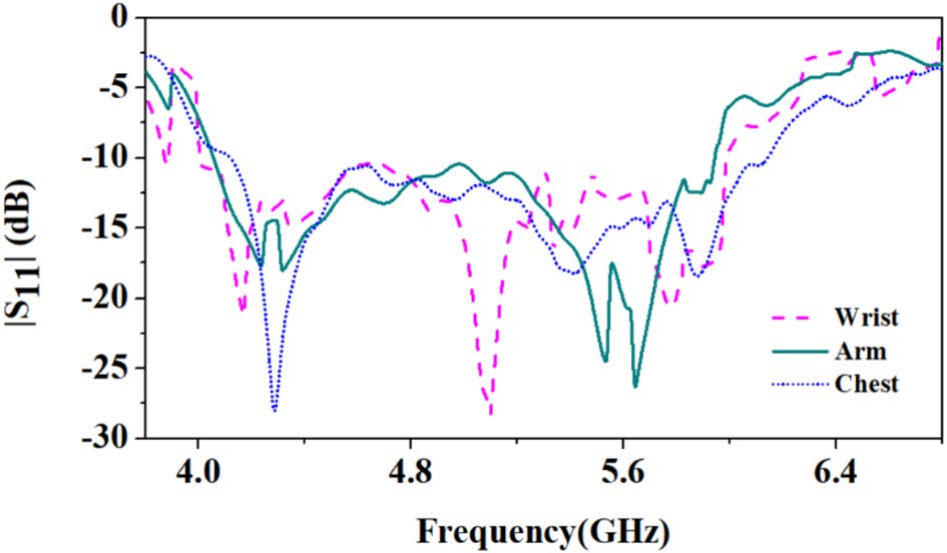
Comparison of reflection coefficients of the proposed antenna when placed on the wrist, arm, and chest.

The presented antenna is fabricated on the denim fabric of dielectric constant 1.72, and its fabricated prototype is shown in Fig. 17. After the fabrication, the performance of the antenna on different parts of the human body (over wearable clothes), such as the wrist, arm, and chest, is evaluated (shown in Fig. 18). The corresponding results are shown in Fig. 19. Bending shifts the resonant frequency to the lower side of the frequency band.

When the antenna is placed on the wrist, it experiences maximum bending, increasing the effective dielectric constant^29^ and shifting the resonant frequency to the lower side (at 4.2 GHz) of the frequency band. When the antenna is placed on the chest, there is negligible shifting in the frequency because of the minimum bending experienced by the antenna due to its compact size over the large chest size.

- **B. Bending analysis of the fabricated prototype in off-body scenario**

**Figure 20:**
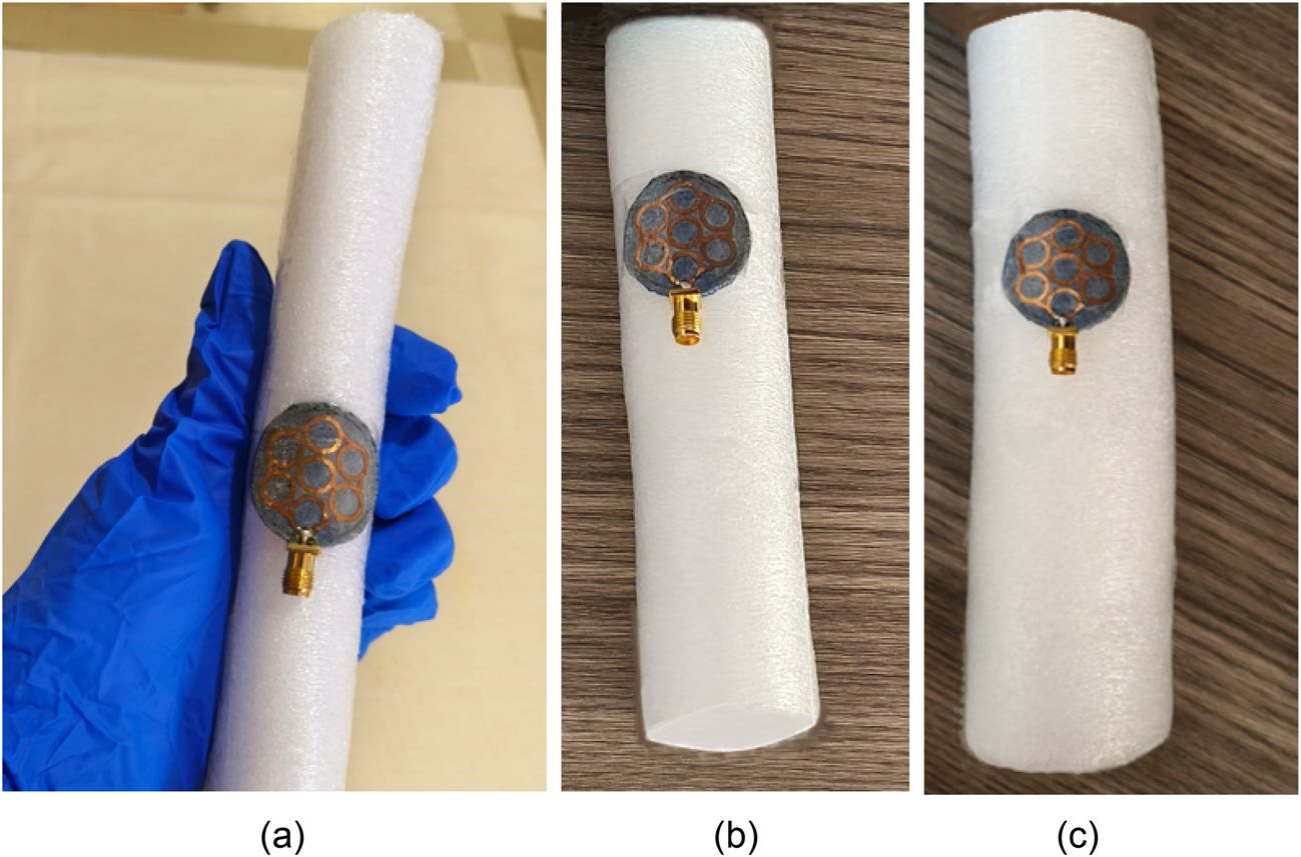
Bending of the proposed antenna on polyurethane foam cylinders of radius: (**a**) 20 mm, (**b**) 40 mm, (**c**) 60 mm.

In this section, the S-parameters of the antenna are presented in different off-body bending scenarios when placed on the polyurethane foam cylinders^30,31^. For bending analysis, the antenna is bent on cylinders with radii of 20 mm, 40 mm, and 60 mm, as shown in Fig. 20, which includes bending less than the minimum bending (20 mm) faced by the antenna (wrist of a newborn baby: 35 mm).

**Figure 21:**
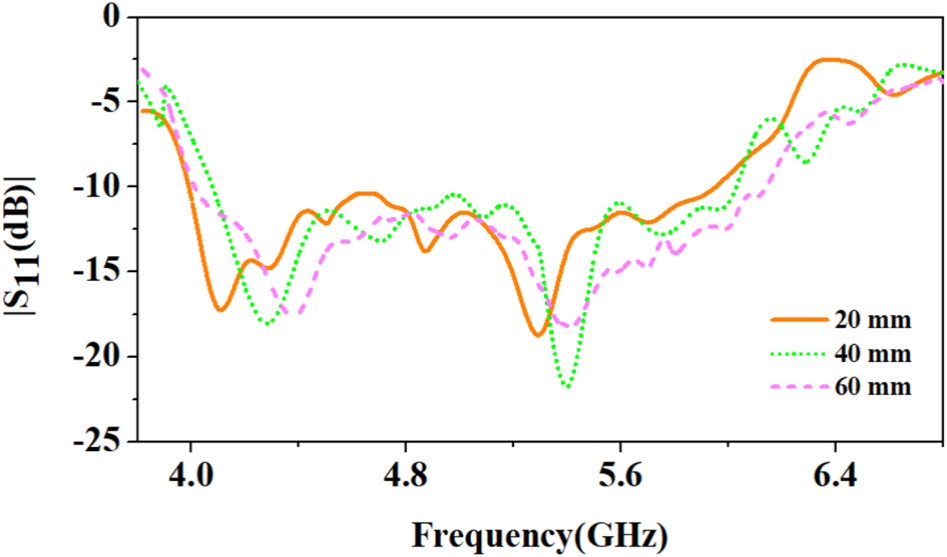
Bending of the proposed antenna on multi-layer arm phantom body model of radius: (**a**) 40 mm, (**b**) 60 mm, (**c**) 80 mm.

Figure 21 shows the reflection coefficients of the antenna for bending radiuses of 20 mm, 40 mm, and 60 mm. It is noticed that the off-body bending shifts both resonances (4.5 GHz and 5.8 GHz) compared to on-body bending, but due to its ultra-wideband behaviour, the antenna effectively covers the reported 5G n79 4.5 GHz band and ISM 5.8 GHz frequency band.

- **C. Far-field measurement**

**Figure 22:**
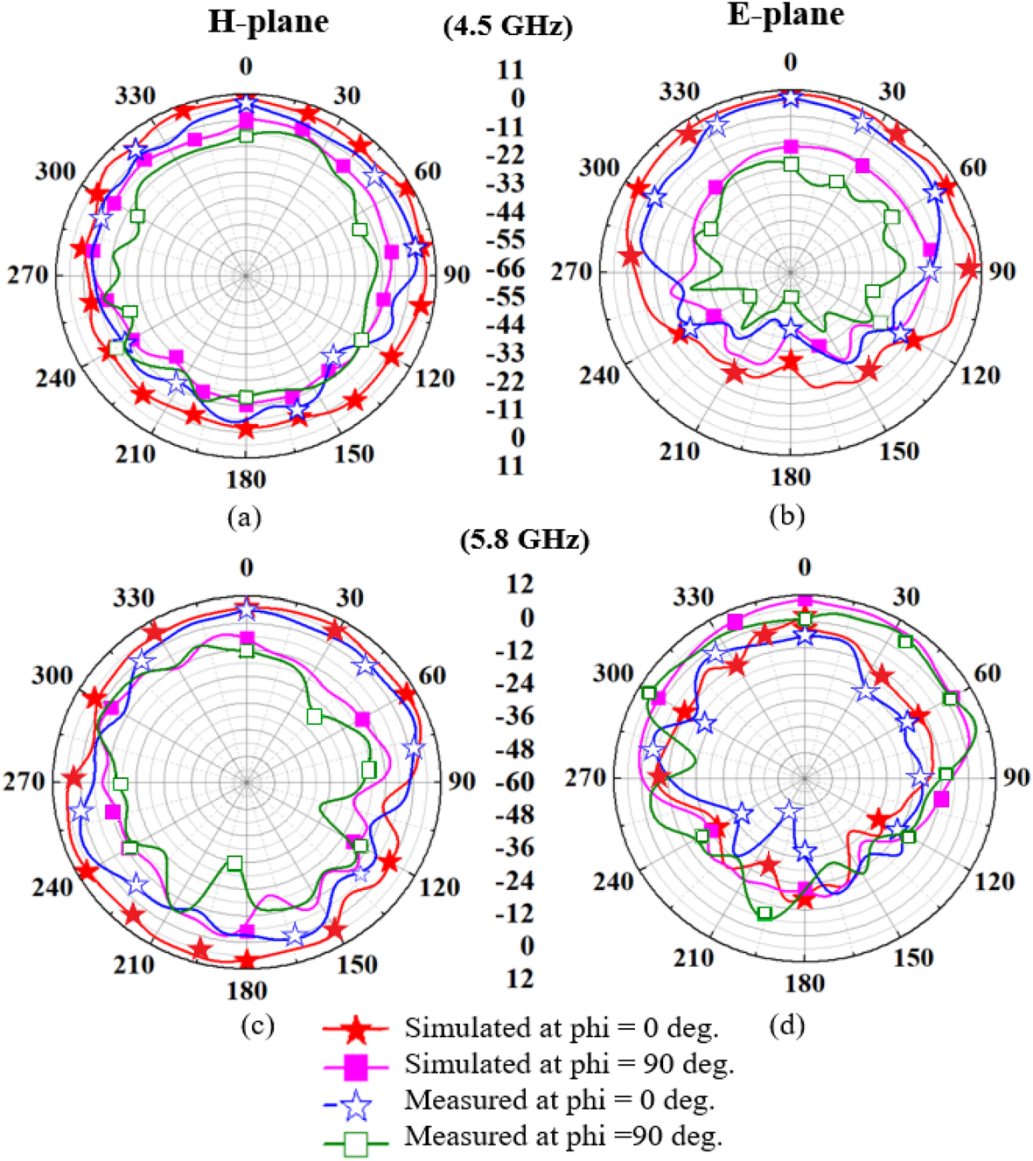
Simulated and measured radiation patterns of the proposed antenna: (**a**) *H*-plane (at 4.5 GHz), (**b**) *E*-plane (at 4.5 GHz), (**c**) *H*-plane (at 5.8 GHz), (**d**) *E*-plane (at 5.8 GHz).

Figure 22 depicts a comparison of simulated and measured *H*- and *E*-plane gain radiation patterns. The simulated and measured peak gains are demonstrated in Table 2. At 4.5 GHz and 5.8 GHz frequencies, radiation patterns in the *H*-plane and *E*-plane at *ϕ* = 0˚ and 90˚ are nearly omnidirectional with multiple closely spaced fluctuations, as shown in Figs. 22(a) − (d). The simulated and measured peak gain values at 4.5 GHz in the *H*-plane (at *ϕ* = 0˚) and in the *E*-plane (at *ϕ* = 0˚) are 8.42 dBi and 9.2 dBi, and 10.5 dBi and 8.9 dBi, respectively. At 5.8 GHz, the simulated and measured peak gain values in the *H*-plane (at *ϕ* = 0˚) and in the *E*-plane (at *ϕ* = 0˚) are 11.1 dBi and 10.6 dBi, and 12.0 dBi and 9.5 dBi**.**

**Table 2:** Simulated and measured peak gain values.

Frequency (GHz)	Simulation (Chest phantom) (dBi)	Measured (On human chest) (dBi)
4.5	10.5	9.2
5.8	12.0	10.7

As we know, biological tissues have different permittivity and conductivity, interact with the antenna’s radiation pattern, and may cause some disturbances^32,33^. So, some ripple-like effects can be seen, when the antenna is operating in the close vicinity of the human body. Since the multi-layer phantom and the real human body differ in size, some variations in the values of gain are noticed in both the reported frequency bands, as shown in Table 2.

- **D. SAR analysis**

**Figure 23:**
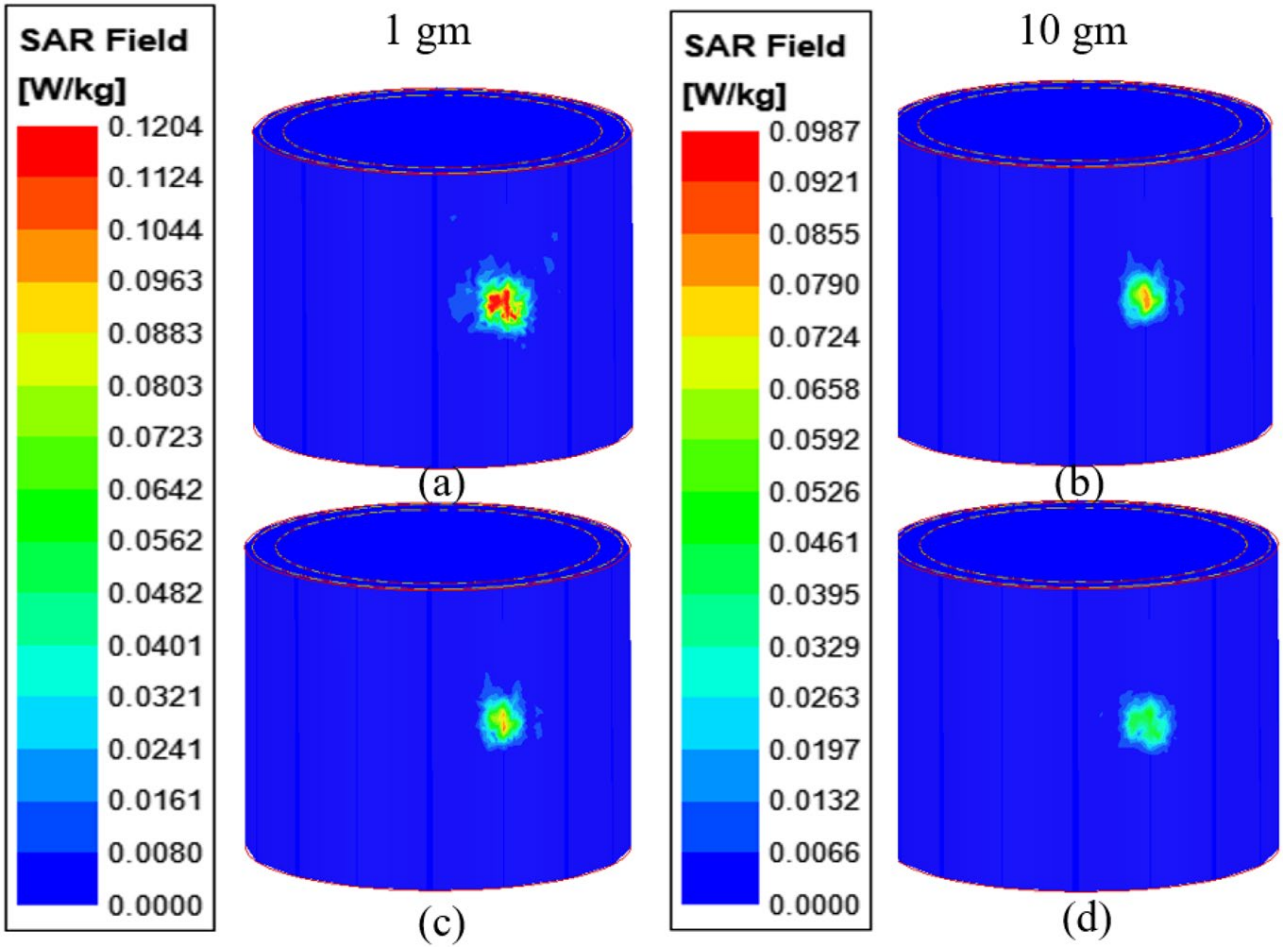
Average SAR distribution in the multilayer tissue model: (**a**) 1 g at 4.5 GHz, (**b**) 10 g at 4.5 GHz, (**c**) 1 g at 5.8 GHz, (**d**) 10 g at 5.8 GHz.

SAR is an important parameter to consider when designing an antenna for use near the human body. SAR provides information about the amount of power absorbed by biological tissues when an antenna works on the human body. According to FCC and ICNIRP standards, the power absorbed by a 1 gm and 10 gm cube of biological tissue should be less than 1.6 W/Kg and 2.0 W/kg, respectively. Figure 23 shows the 1 gm/10 gm SAR values at 4.5 GHz and 5.8 GHz. When 1 W of input power is applied to the antenna, the 1 gm/10 gm SAR values at 4.5 GHz and 5.8 GHz are 0.12/0.098 W/kg and 0.11/0.082 W/kg.

Table 3 shows that the proposed wearable textile antenna has very low SAR values, indicating that it can be used safely in wearable applications. In this case, an input power of 1 W is used to calculate SAR values, but the power required for wearable applications is milliwatts, so SAR values in the milliwatt range will be much lower than 1 W. Hence, it can be concluded that the presented antenna is safe for long-term use in both on- and off-body communications.

- **E. Link budget analysis (for on-body and off-body communication)**

**Table 3:** Maximum average SAR values (1 gm/10 gm) at 1 W of input power.

Frequency (GHz)	Average SAR (On chest phantom)	
	1 g	10 g
4.5	0.12	0.098
5.8	0.11	0.082

**Figure 24:**
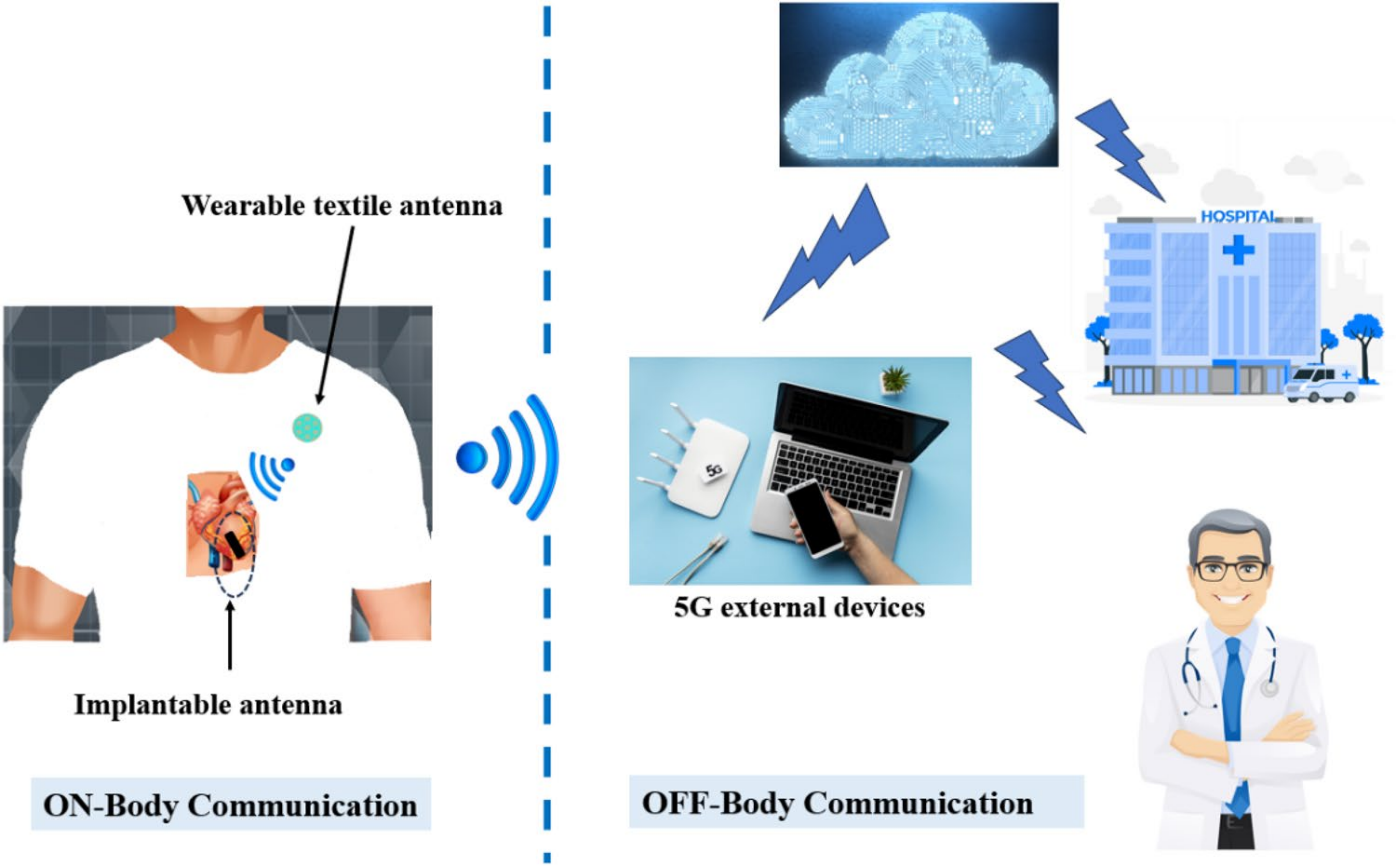
Schematic of on-body communication and off-body communication scenario (image is adapted from^34^).

**Figure 25:**
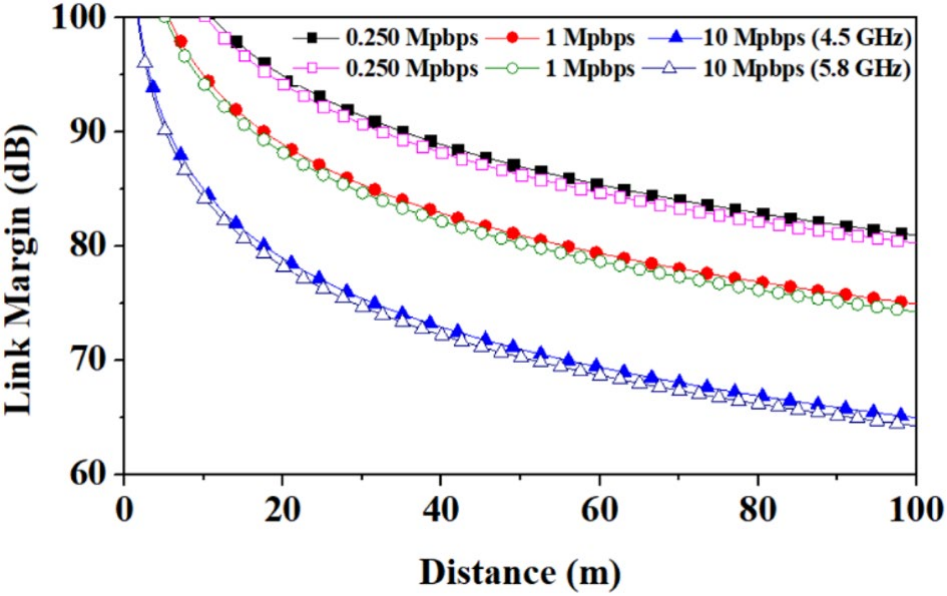
Link margin for off-body communication: Between outside body and wearable (proposed) antennas at 5G n79 4.5 GHz frequency band.

The proposed work shows the communication of the proposed wearable antenna to the implantable antenna and outside-body antenna for on-body and off-body communications, respectively, as shown in Fig. 24. The link margin for off-body communication is plotted in Fig. 25, in this case communication link is established between an external monopole 5G antenna (gain of 2.15 dBi) and the proposed wearable antenna.

Whereas, an implantable antenna proposed in reference^35^ has the gain of −21.8 dBi and is considered the receiving antenna for on-body communication in the 5.8 GHz frequency band. A communication link budget is calculated to send real-time biological signals from the implantable medical device to an external device. The Friis transmission equation is used to calculate the link budget, as explained in^36,37^. The link margin equations from the reference^38^ are used to plot the graph of the link margin versus the transmitter–receiver (*T*_*x*_–*R*_*x*_) distance. Table 4 is used to estimate the link margin at the reported frequency bands (4.5/5.8 GHz), and the graphs between the link margin and telemetry (*T*_*x*_–*R*_*x*_) distance.

**Table 4:** Link budget parameters for on-body and off-body communication.

*Transmitter*			
	Frequency (GHz)		4.5/5.8
*G*_*t*_	Antenna gain (dBi)		10.5/12.0
*P*_*t*_	Transmitter power (dBm)		16
	EIRP (dBm)		26.5/28.0
*ν*	Linear axial ratio		3.1/11.0
*e*_*p*_ _*(dB)*_	Polarization loss	*α* = 0° = 180°	0.249/0.37
		*α* = 90° = 270°	0.73/0.54
*Receiver*			
*G*_*r*_	Receiver antenna gain (dBi) (Outside-body/implantable)		2.15/−21.8
*T*_*o*_	Ambient temperature (K)		293
	Boltzmann constant		1.38E − 23
*N*_*o*_	Noise power density (dB/Hz)		−203.9
*Signal quality*			
*B*_*r*_	Bit rate (Mbps)		0.250, 1, 10
*E*_*b*_/*N*_*o*_	Ideal PSK (dB)		9.6
*G*_*c*_	Coding gain (dB)		0
*G*_*d*_	Fixing deterioration (dB)		2.5

**Figure 26:**
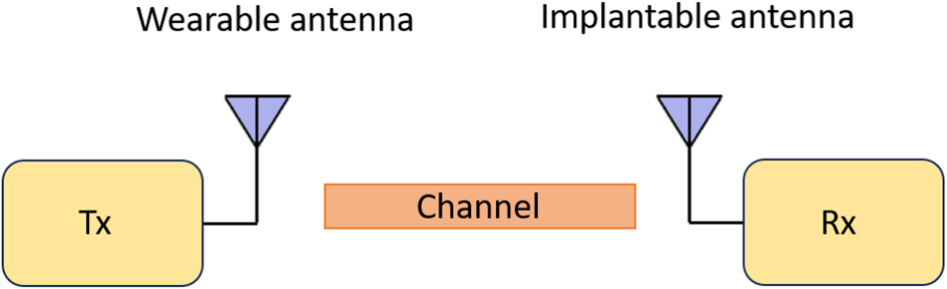
Block diagram for the link budget calculation in the on-body communication: Between implantable and wearable (proposed) antennas at ISM 5.8 GHz frequency band.

**Figure 27:**
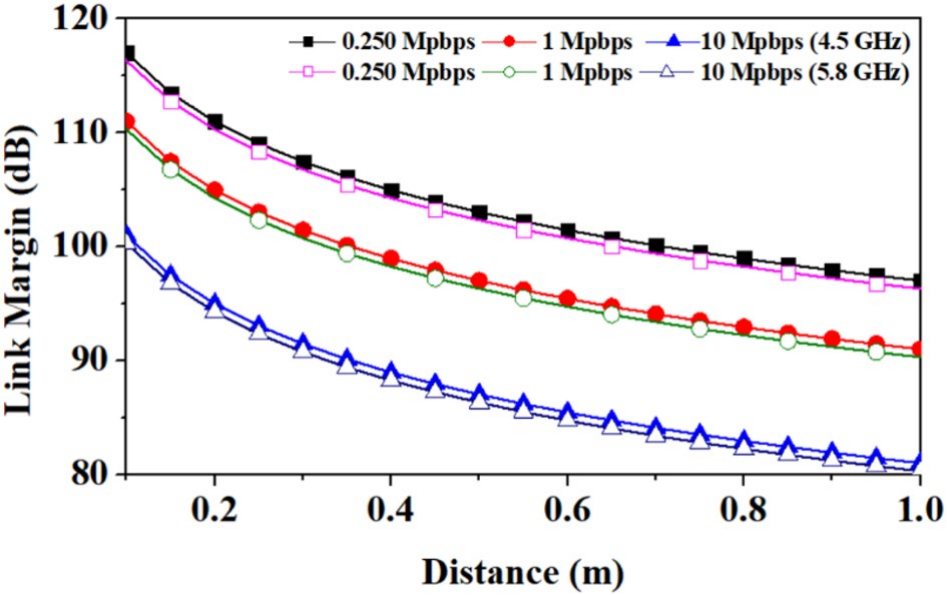
Link margin for on-body communication: Between implantable and wearable (proposed) antennas at ISM 5.8 GHz frequency band.

The link margin values at different data rates of 0.250 Mbps, 1 Mbps, and 10 Mbps are evaluated. The link margin set-up is taken from reference^39^ is shown in Fig. 26 for on-body communication, and Fig. 27 depicts the corresponding results. Figs. 25 and 27 show the communication link for the distance of 100 m and 1.0 m with link margin values of 65 dB and 80 dB for off-body and on-body (implant-to-wearable) communication, respectively.

**Figure 28:**
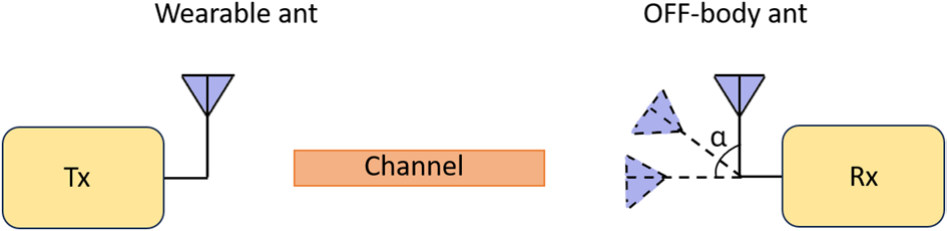
Link margin for off-body communication: Between outside body and wearable (proposed) antenna at 5G n79 4.5 GHz frequency band (when polarization mismatch (*e*_*p*_) is considered).

**Figure 29:**
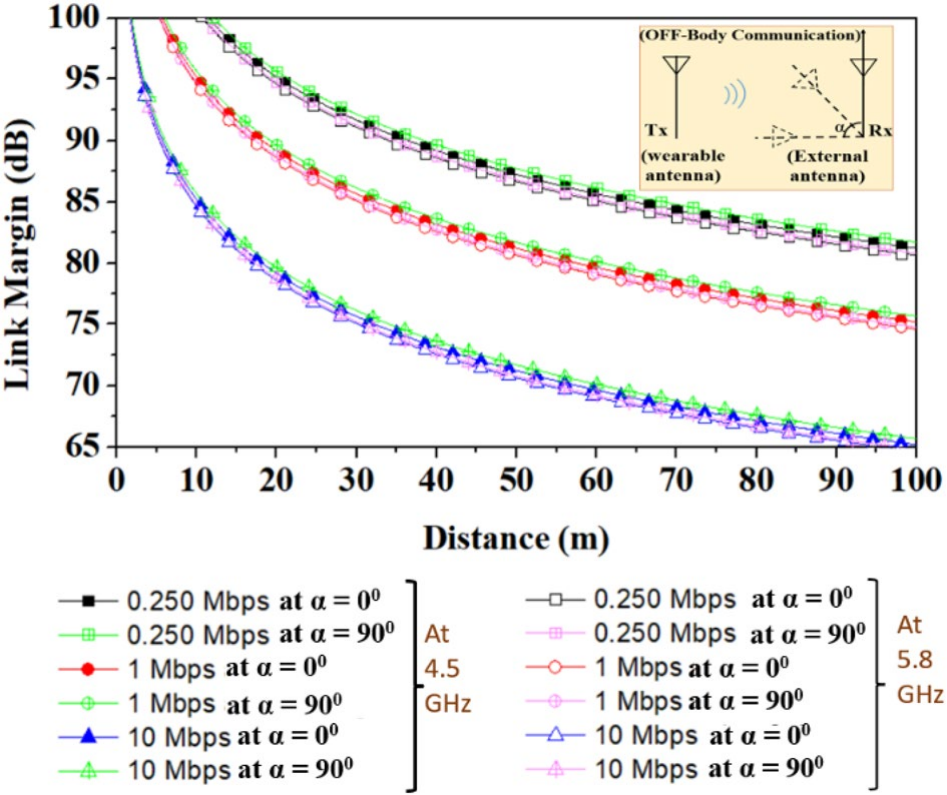
Link margin for off-body communication: Between outside body and wearable (proposed) antennas at 5G n79 4.5 GHz frequency band (when polarization mismatch (*e*_*p*_) is considered).

On the other hand, when we have considered polarization mismatch losses, as shown in Fig. 28, the antenna can efficiently communicate at 10 Mbps data rate, at 5.8 GHz, up to 99.2 m and 97 m, for *α* = 0° = 180° and *α* = 90° = 270°, respectively. Thus, the proposed antenna can communicate over a reasonable distance even if polarization mismatches occur, as illustrated in Fig. 29. The polarization loss at different angles of the external antenna (*α*) is calculated from the equation given in^40^.

Hence, it can be concluded that the proposed antenna communicates effectively with both off-body and implantable antennas.

## Conclusion

A dual-band wearable textile antenna is proposed for 5G-n79 and ISM 5.8 GHz frequency band applications. The designed antenna has a high gain (10.5 dBi and 12.0 dBi) and an ultrawide impedance bandwidth. Bending analysis is done on the actual human body (over wearable clothes) to determine the usefulness of the proposed antenna in various bending scenarios. Due to its large impedance bandwidth, the antenna can operate in the reported frequency bands even when detuning occurs. The measured results are consistent with the simulation results, and the 1 gm and 10 gm SAR values are significantly lower than the standard values. Thus, the proposed antenna has ultrawide impedance bandwidths, good gain, and lower SAR values, making it suitable for 5G n79 4.5 GHz and ISM 5.8 GHz off-body and on-body communications, respectively.

## Data Availability

There is no separate dataset generated in this study. All the data is mentioned in the manuscript.
